# Integrated miRNA-mRNA analysis in the habenula nuclei of mice intravenously self-administering nicotine

**DOI:** 10.1038/srep12909

**Published:** 2015-08-11

**Authors:** Sangjoon Lee, Jiwan Woo, Yong Sik Kim, Heh-In Im

**Affiliations:** 1 Center for Neuroscience, Brain Science Institute; 2 Research Animal Resource Center, Korea Institute of Science and Technology, Hwarangno 14-gil 5, Seongbuk-gu, Seoul, Republic of Korea; 3 Neuroscience Program, Korea University of Science and Technology, 217 Gajungro, Yuseong-gu, Daejeon, Republic of Korea; 4 Seoul National University College of Medicine, Seoul, Republic of Korea

## Abstract

A considerable amount of evidence suggests that microRNAs (miRNAs) play crucial roles in the neuroadaptation of drug addiction. Habenula (Hb), one of the critical brain regions involved in reward and addiction, can be divided into two anatomically and transcriptionally distinct regions: medial habenula (MHb) and lateral habenula (LHb) nuclei. However, very few studies have compared the functional roles of these regions. Here, by using mirConnX integrator and KEGG pathway mapping, we simultaneously analysed the differential expression patterns of miRNAs and messenger RNA (mRNA) within MHb and LHb under nicotine addiction. Significantly altered miRNAs and mRNAs were found in the Hb of mice intravenously self-administering nicotine. Interestingly, some miRNAs were oppositely regulated between the MHb and the LHb, and their potential targets included various genes of cell signalling pathways related to the degeneration of fasciculus retroflexus (FR). This study provides an improved insight into the differential regulation of habenular transcripts in nicotine addiction, as well as the potential functions of miRNAs in several biological pathways involved in the nicotine addiction.

MicroRNAs (miRNA) are non-coding RNAs that regulate numerous biological functions in various organisms through degradation of mRNAs and repression of translation. By binding with complementary regions of mRNA molecules, miRNAs destabilize mRNAs and decrease the efficiency of protein expression. Recent studies revealed that drug addiction and its subsequent neuroadaptive changes could be regulated by miRNAs within the brain^1^.

Tobacco dependence has a complex genetic trait, where greater than 50% risk of developing dependence is attributable to genetic factors^2^. Nicotine, the major psychoactive component in tobacco responsible for dependence, functions in the brain through neuronal nicotinic acetylcholine receptors (nAChRs). These nicotinic receptor subtypes are intermediately expressed in the mesolimbic dopamine tract. However, high number of nAChRs are concentrated in the medial habenula (MHb) and in the interpeduncular nucleus (IPN), which together comprise a major cholinergic tract in the mammalian brain^3^,^4^,^5^,^6^.

The habenular complex that links the forebrain and the midbrain structures could be subdivided into the medial (MHb) and the lateral (LHb) nuclei. With regard to the LHb, two main projections to the ventral tegmental area (VTA) were studied; one is direct glutamatergic excitatory projection and the other is indirect projection to VTA passing the rostromedial tegmental nucleus (RMTg) region. Because the RMTg has GABAergic inhibitory projection to the VTA, these two projections complementarily cooperate to regulate the neuronal activity of the VTA. Many studies of the LHb have implied its functions in both aversion- and reward-related behaviors, while its projection to the nucleus accumbens (NAcc) has been controversial because the functions of the shell and the core regions of the NAcc have not been clearly resolved^7^,^8^. The VTA regulation has also been studied with regard to the MHb. It is noteworthy that the projection from the MHb to the IPN, which is called fasciculus retroflexus (FR), has been identified to play a major role in nicotine addiction. IPN sends its GABAergic inhibitory projection to the VTA, and the excitatory signals from the MHb to the IPN via glutamate and acetylcholine signalling could inhibit the VTA functionally^9^,^10^,^11^.

Accordingly, previous studies on the function of the Hb complex under the state of nicotine addiction have focused on how nAChRs play a role in these circuitries. However, molecular dynamics other than nAChR functions have been undervalued and still poorly understood. In this study, the expression changes of miRNAs and mRNAs in both the MHb and the LHb nuclei were analysed using microarrays after the intravenous self-administration of nicotine. Microarray experiments have been widely used for the systemic analysis of various target molecules, including miRNAs or mRNAs, by measuring the intensity of their expressions. With multiple microarrays from different experimental groups, detailed molecular profiles can be extracted and comparatively analysed. Therefore, in this study, the integrated miRNA-mRNA expression analysis was adopted to compare the transcriptional networks of the MHb versus the LHb nuclei and show the various molecular regulations and the functional implications of the Hb in nicotine addiction.

## Results

### Mice intravenously self-administering nicotine showed the general drug-seeking behavior

**Figure 1:**
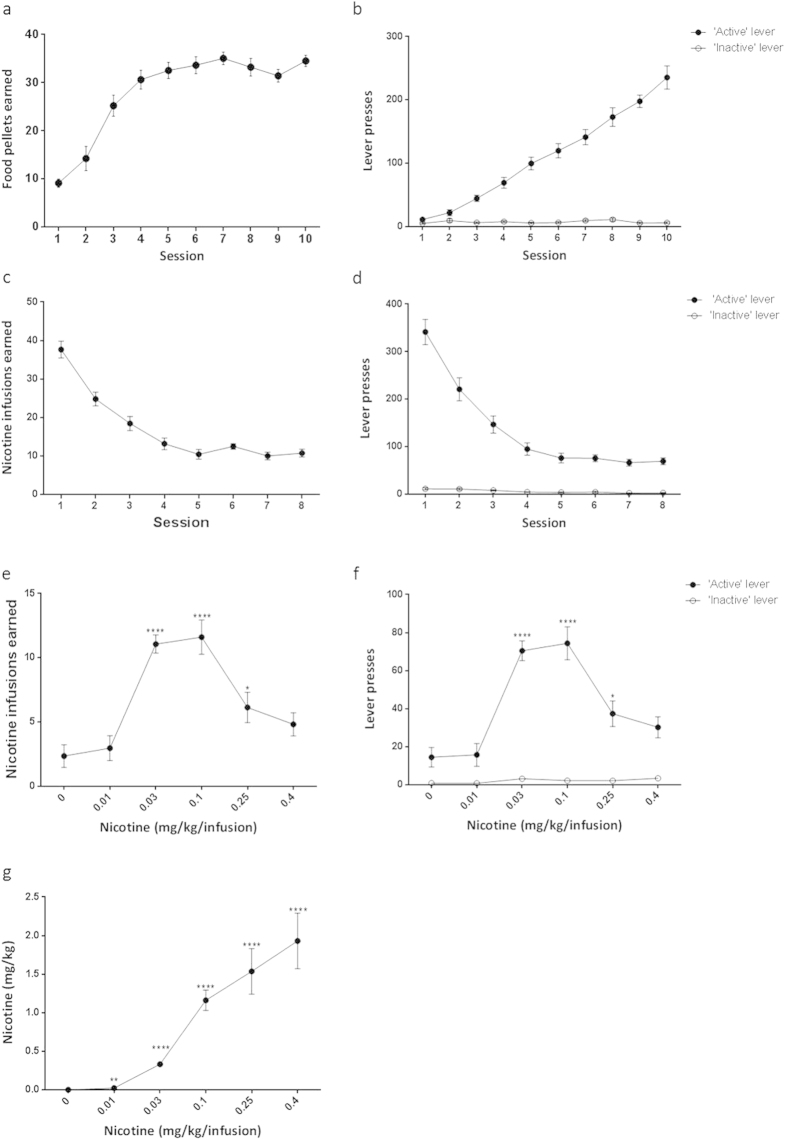
Mice intravenously self-administering nicotine showed the general drug-seeking behaviour. Food pellets and intravenous nicotine infusions were earned during the self-administration sessions. (**a**,**b**) Mice pressed ‘active’ lever to receive food pellets during 1 h sessions. (**c**,**d**) Number of nicotine infusions earned and ‘active’ lever to receive nicotine infusion (0.03 mg/kg per infusion). (**e**,**f**) Dose-response test of nicotine SA showed inverted U-shaped dose-response curve with the highest intake at the 0.1 mg/kg per infusion. Each dose was tested for 5 days and the reinforcement numbers of the final 3 days were averaged. (**g**) Total quantity of nicotine infused at each dose was calculated. All data are presented as mean ± SEM of nicotine infusions or lever presses (* *P*< 0.05, **P < 0.01, ****P < 0.0001).

Prior to the nicotine infusion, mice underwent 10 days of food training in the operant self-administration (SA) chamber during which the mice learned to obtain the food pellet reward when they pressed the ‘active’ lever instead of the ‘inactive’ lever (Fig. 1a,b). Subsequently, the reward was changed from the food pellets to the nicotine infusion (0.03 mg/kg per infusion). Mice consumed ~40 nicotine rewards during their first SA session, but rapidly adjusted their level of responding over the next 2–3 sessions to obtain ~11 rewards during the consecutive 1 h session (Fig. 1c). Mice stably pressed the ‘active’ lever significantly more than ‘inactive’ lever throughout the nicotine infusion experiment (Fig. 1d). When the unit dose of nicotine available was varied (0, 0.01, 0.25, 0.1 and 0.4 mg/kg per infusion), mice received nicotine infusions according to an inverted U-shaped dose-response curve (Fig. 1e). Compared to the active lever, inactive lever responses remained low across all nicotine doses (Fig. 1f). Interestingly, mice continued to increase drug intake throughout all unit doses of nicotine (0.01, 0.25, 0.1 and 0.4 mg/kg per infusion) (Fig. 1g).

### Microarray profiling of the habenular transcripts showed differentially altered expressions in mice intravenously self-administering nicotine

**Figure 2:**
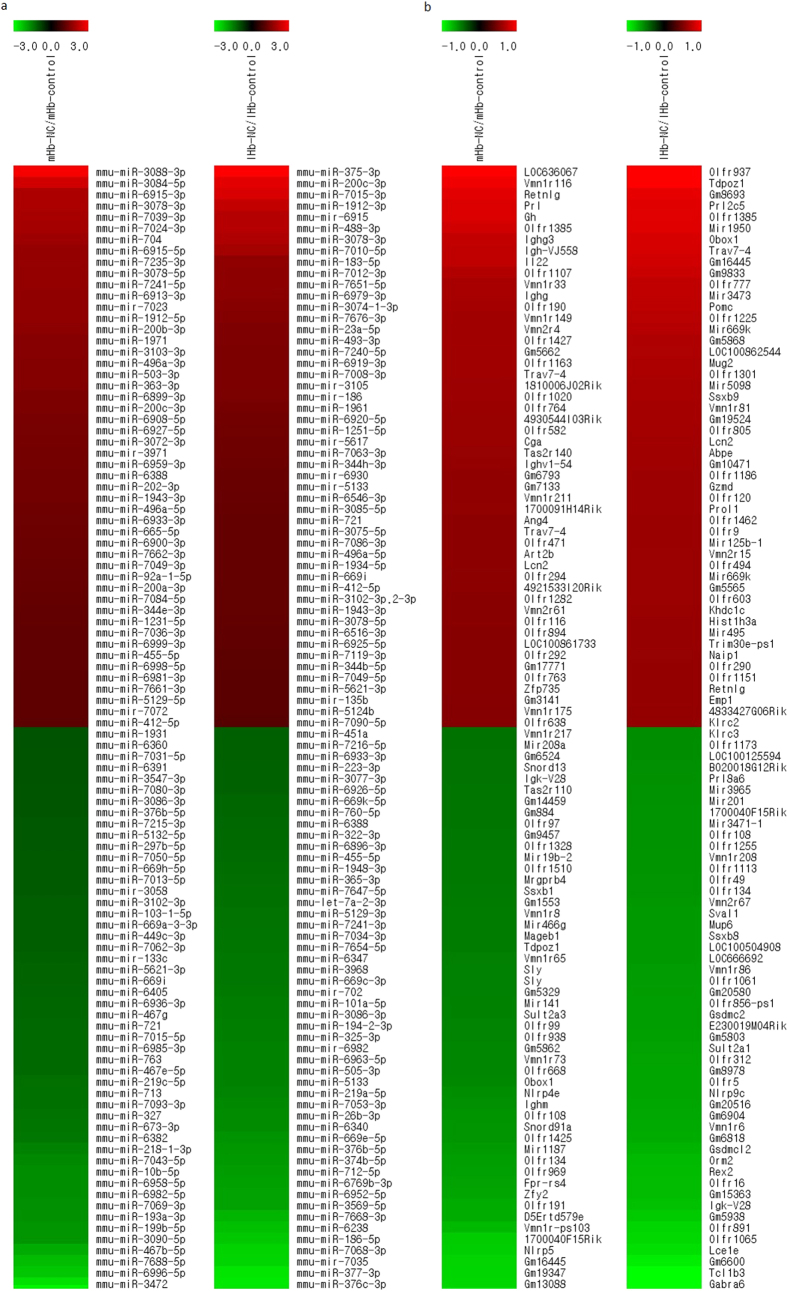
Expression profiles in the habenula of mice intravenously self-administering nicotine and age-matched drug naïve control. Heatmaps of the most prominently altered transcripts selected from the microarray data. (**a**) miRNA expressions in MHb (left) and LHb (right) comparing the intravenous nicotine SA group (NC) and age-matched drug naïve control group. (**b**) mRNA expressions in MHb (left) and LHb (right) comparing the intravenous nicotine SA group (NC) and age-matched drug naïve control group. Red denotes increased expression level, whereas green denotes decreased expression level.

**Figure 3:**
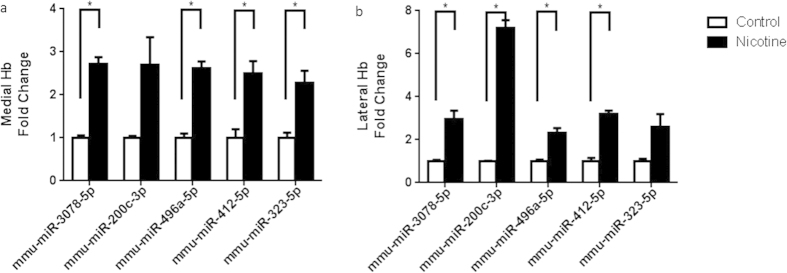
Quantitative comparison of miRNA expression in the habenula of mice intravenously self-administering nicotine and age-matched drug naïve control. Five miRNAs selected from the list of altered miRNAs (mmu-miR-3078-5p, mmu-miR-200c-3p, mmu-miR-496a-5p, mmu-miR-412-5p, mmu-miR-323-5p) were additionally confirmed by qRT-PCR of the intravenous nicotine SA group and the age-matched drug naïve control group (*P < 0.05).

The MHb and the LHb exhibited differential expression profiles of numerous miRNAs and mRNAs after intravenous nicotine SA (Fig. 2a,b). Among 30434 miRNAs analysed, a total of 44 miRNAs were found to be significantly altered. In the MHb, 8 miRNAs increased more than two-fold and 7 miRNAs decreased under half compared to the drug-naïve group. In the LHb, 10 miRNAs increased more than two-fold, and 8 miRNAs decreased under half. Five miRNAs increased in both the MHb and the LHb. It is interesting to note that 18 miRNAs were altered oppositely with different degrees in the MHb and the LHb (Table 1). The alterations of some miRNAs were additionally confirmed by qRT-PCR assay (Fig. 3a,b). Alterations of mmu-miR-3078-5p, mmu-miR-200c-3p, mmu-miR-496a-5p, mmu-miR-412-5p, and mmu-miR-323-5p were tested in the MHb and the LHb with the same samples used in the microarrays, and their altered expression patterns were well-matched with the array data (Fig. 3).

### A mirConnX analysis of the miRNA-mRNA network identified various targets of forty-four miRNAs altered by the mice self-administering nicotine

**Figure 4:**
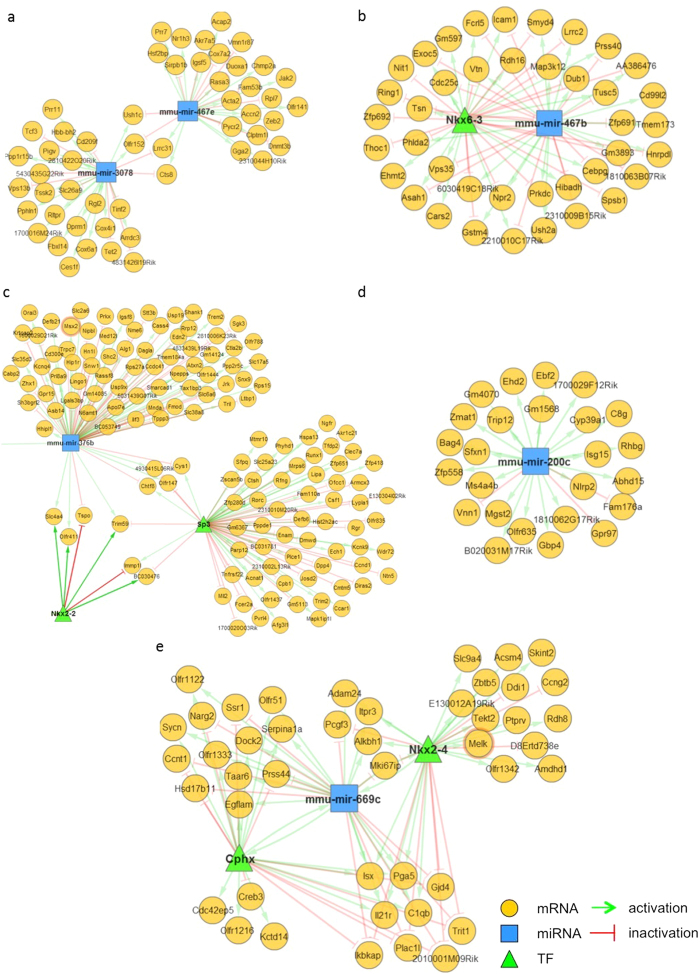
miRNA-mRNA interaction networks generated by mirConnX. Altered miRNAs were analyzed in various patterns including (**a**) miRNA-miRNA network, (**b**,**c**,**e**) miRNA-transcription factor network and (**d**) single miRNA network. The interactions among molecules are shown by green arrows and red lines, which represents activation and repression, respectively.

To confirm the correlations between the array of the miRNAs and the mRNAs, 44 altered miRNAs were analysed using the mirConnX web interface (http://mirconnx.csb.pitt.edu) with the Pearson’s correlation as a measurement of association. The representative networks of miRNA-mRNA interactions were derived from the miRNA target predictions, and the interaction patterns were analysed within the total network (Fig. 4). It is noteworthy that both mmu-miR-3078 and mmu-miR-467e were associated within a single network regulating four common targets, *Ush1c*, *Olfr152*, *Lrrc31* and *Cts8*. (Fig. 4a). In addition, mmu-miR-669c, mmu-miR-467b and mmu-miR-376b were associated with other transcription factors for the regulation of the common targets within each regulation network.

### Altered miRNAs have putative targets overlapping with the mRNA targets related to the various functions of the MHb and the LHb

**Figure 5:**
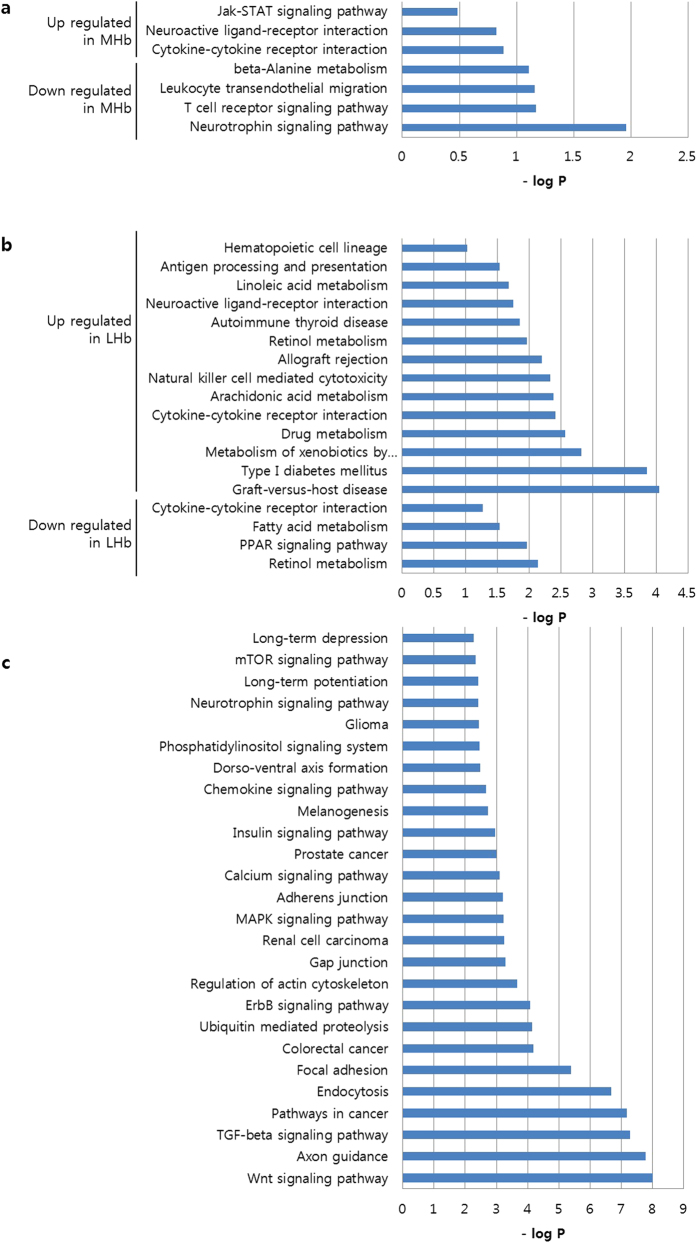
Altered KEGG pathway targeted by the nicotine-responsive miRNAs. KEGG pathways targeted by altered 44 miRNAs were analyzed based on the mRNAs targeted by altered miRNAs. KEGG pathways targeted by altered miRNAs within (**a**) MHb and (**b**) LHb. (**c**) Altered pathways targeted by oppositely altered miRNA are shown. The vertical axis is the pathway categories, and the horizontal axis is the enrichment of pathways.

The 44 miRNAs could target ~3000 genes based only on the sequence complementarity in the miRNA target database such as DIANA database and microRNA.org. Among these genes, 615 mRNAs were found to be altered in the mRNA microarray profiling. These mRNAs had reverse patterns of alteration with the corresponding 44 miRNAs, which could be interpreted as the repression of the mRNAs by the miRNAs (Suppl. Table S8). It is notable that there were 10 oppositely regulated mRNAs between the MHb and the LHb whose corresponding miRNAs were also oppositely altered (Table 2). Next, various categories of KEGG pathways were analysed through DAVID functional annotation tool (Fig. 5). Among the miRNA targets within the MHb, the neurotrophin signalling pathway and the neuroactive ligand-receptor interaction were found to be altered by intravenous nicotine SA. Among the miRNA targets within the LHb, various pathways including retinol metabolism and drug metabolism were found to be changed. For the analysis of the targets of oppositely regulated miRNAs in the MHb versus the LHb, the MAPK signalling pathway and the calcium signalling pathway were found to be altered by intravenous nicotine SA.

## Discussion

This is the first study to show the characteristic expression patterns of transcripts (miRNAs and mRNAs) for the MHb and the LHb in a mouse model of nicotine addiction. We analysed three patterns (up-regulated, down-regulated and oppositely altered) of the miRNA expression profiles in the MHb and the LHb compared to the drug-naïve group for the functional study of nicotine addiction. By exploring the three patterns of miRNA expression profiles, we found significant differences in terms of gene ontology features. In addition, we found that the expression of up-regulated miRNAs in the LHb showed more dramatic changes than the changes in the MHb. Furthermore, we discovered an interesting aspect of the oppositely altered miRNAs in the MHb versus the LHb showing that the targeted mRNA networks are mainly involved in the development of nicotine addiction.

At present, the MHb is known to regulate various behavioral functions, including stress responses, depression and addiction^12^. The function of MHb in nicotine addiction has been elucidated from the perspectives of the neurotoxicity and the nAChR functions. A previous report on nicotine-induced neurotoxicity revealed that the neuronal projection from the MHb to the IPN, which is called FR, is degenerated by nicotine treatment^13^. Consequently, the nicotine-induced FR degeneration has been interpreted as a loss of forebrain control by drug abuse, possibly leading to the addiction of the nicotine^14^. Additionally, several recent studies have revealed that highly localised nAChR expression in the MHb regulates the condition of nicotine addiction^15^,^16^. According to our KEGG pathway analyses, altered miRNA-mRNA networks including 15 miRNAs and 156 mRNAs in the MHb appeared to regulate immune-response pathways including cytokine-cytokine receptor interaction and T cell receptor signalling pathways and neuroactive ligand-receptor interaction between GABA and GABA receptor (Suppl. Figs S1–S3). In general, it has been suggested that the increased incidence of various diseases in smokers may be due to altered immune responses rising from the chronic inhalation of chemicals (e.g. nicotine) in cigarette smoke^17^. In this respect, nicotine is known to induce T-cell anergy and immunosuppression^18^. Therefore, the alteration of these pathways observed in our study could be interpreted as missing links between the nicotine neurotoxicity and the signalling pathway of nicotine which were found to be regulated by the nicotine responsive miRNA-mRNA in our study^19^,^20^. In this regard, it is also possible that mmu-miR-200c-3p, which was up-regulated in the MHb by intravenous nicotine SA, possibly regulates phospholipase C, gamma 1 (Plcg1), which is shown to regulate immune-response pathways, namely the T-cell receptor signalling pathway and neurotrophin signalling pathway (Suppl. Figs S2 and S3).

Multiple studies of the LHb nucleus have revealed its regulatory function on the dopaminergic pathways of reward circuit in the brain, particularly concerning the negative prediction errors in the behavioral studies^7^,^8^,^21^. In intravenous nicotine SA, negative states of addiction could be induced in the brain because the mice experienced both reinforcement and withdrawal during the period of nicotine SA. In KEGG analyses of the LHb mRNA profile, the gene regulation induced by the PPAR signalling was involved with nicotine addiction (Suppl. Fig. S4). A previous report found that nicotine withdrawal could be modulated by the PPARα ligands and the PPARα agonists^22^, while another study using the inhibitor of PPARγ reported that the nicotine-induced gene expression could be regulated by the PPAR signalling pathway through upregulation of CD36 signaling^23^.

Opposite alteration patterns of the miRNA-mRNA networks between the MHb and the LHb also provided an interesting interpretation regarding the role of the Hb in mice intravenously self-administering nicotine. Our data revealed that several KEGG pathways, including MAPK signalling, calcium signalling and neurotrophin signalling, were oppositely regulated between the MHb and the LHb by the oppositely altered miRNAs (Suppl. Figs S5 and S6). It is noteworthy that these pathways have been studied in respect to the effects of nicotine^24^,^25^,^26^. On the other hand, mmu-miR-467c-3p is also oppositely regulated in the MHb *vs* the LHb and targets the brain-derived neurotrophic factor (Bdnf), a signalling protein in the central nervous system that is well-known as a positive regulator of substance abuse such as cocaine^27^. In addition, mmu-miR-721 was found to target the mitogen-activated protein kinase 1 (Mapk1), a well-known kinase acting on the intersecting point of various biochemical signalling pathways including differentiation, transcription and development. It is noteworthy that nuclear factor-kB (NF-kB) and cyclic AMP response element binding protein (CREB) were shown to function in downstream of MAPK signalling (Suppl. Fig. S5) and these factors have been extensively studied in the drug abuse and addiction^28^. Furthermore, mmu-miR-669c-3p regulated the sortilin 1 (Sort1), one of the receptors involved in cell-death signalling, and this regulation may underlie the mechanisms of FR degeneration^29^. Finally, all these targets—Bdnf, NF-kB, CREB and Sort1—were critical regulators of the neurotrophin pathway, and various aspects of the relationship between nicotine addiction and the neurotrophin pathway have been studied in dozens of publications especially dealing with tyrosine kinase (Trk) receptor A, postnatal rat hippocampus and Trk receptor signalling^30^,^31^,^32^.

Altogether, the bioinformatic analysis of the habenular miRNA-mRNA network in mice intravenously self-administering nicotine revealed several useful interpretations about the role of Hb in the nicotine addiction and the neuroadaptive changes by nicotine. In future, numerous *in vivo* experiments will be required to elucidate these interrelations linking those biological processes and miRNA-mRNA networks.

## Methods

### Animals

Seven-week old C57BL/6 mice were purchased from Daehan Biolink (Chungbuk, Korea). For both the nicotine-administering group and the age-matched drug naïve control group, mice were individually housed for 12 hours with a reversed light-dark cycle in a laboratory breeding room at the Korea Institute of Science and Technology (KIST). For the nicotine-administering group, water was freely available but foods were mildly restricted throughout the test (about 85–90% of their free-feeding body weight). For age-matched drug naïve control group, water and foods were provided ad libitum. Nicotine SA was conducted in the operant SA chamber enclosed by the sound attenuating cubicle (MED-307A-CT-D1, Med Associates, Inc., St. Albans, USA) in the dark room. All procedures regarding the use and the handling of the animals were conducted as approved by the Institutional Animal Care and Use Committee of the KIST.

### Intravenous self-administration procedure

#### Surgery

Operant nicotine SA for mice was performed according to the method described previously^33^. After completing the ten-day food training with the food pellets in the SA chamber, the mice were anaesthetised by intraperitoneally injecting a mixture of ketamine (120 mg/kg) and xylazine (0.6 mg/kg). Then a catheter was implanted in the jugular vein of the mice and the end of the catheter was placed on the backs of the mice. To prevent infection, instruments used in the surgery were sterilised and the mice were injected daily with gentamycin if necessary.

#### Apparatus

Operant chambers (29.5 cm × 32.5 cm × 23.5 cm) with two retractable levers were used in the tests. The left lever was used as an active lever to confer the reward. Cue light was located above the lever and programmed to be turned on when the lever was pressed correctly. For nicotine infusion, nicotine syringe pumps were connected to the outlet on the backs of the mice via metal spring-covered tubes. The operant chambers were maintained and controlled by MED-PC software (Med Associates).

#### Procedure

Mice were trained to discriminate between the levers and to press the active lever instead of the inactive lever in order to deliver the food reward in the training program prior to the SA of nicotine. The fixed-ratio (FR) was escalated from FR1 to FR5 gradually when the mice received more than 25 food pellets for two consecutive days. After finishing the FR5 food training, the mice had intravenous catheter surgery into the right jugular vein to allow for the nicotine delivery, followed by three days of recovery. At the nicotine SA session, the mice obtained a 0.03 mL nicotine solution as a reward for every active lever press, with FR5 (delivered over three seconds) in a one-hour session. When they were infused with the nicotine solution, a cue light above the active lever was turned on for 20 seconds. When the mice showed a stable infusion in the unit doses of 0.03 mg/kg per infusion, 0.1 mg/kg per infusion was tested and used as a ‘training dose’ between the change of each dose. Dose-response test was done using unit doses of 0, 0.01, 0.25 and 0.4 mg/kg per infusion, with each dose tested over five days and the average of the final three days calculated for the reward number. A latin-square cross-over design was used in the test to control any effect of test order. After dose-response test, 0.03 mg/kg per infusion dose of nicotine was infused to the mice during additional 10 days for optimal induction of nicotine addiction prior to sacrifice. Nicotine solutions were prepared with saline and (-)-nicotine hydrogen tartrate salt (Sigma, St. Louis, MO, USA).

### RNA extraction from the MHb and the LHb and microarray experiments

Mice were decapitated on the next day of the final SA of nicotine. Frozen whole brains were cut into 100 μm slices with the coronal plane on a cryostat (CM3050S, Leica) at −20 °C. The MHb and the LHb were micro-dissected separately (Suppl. Fig. S7), and the total RNA was isolated using the RNA STAT-60 (Amsbio, Abingdon, UK) for the microarray experiments according to the manufacturer’s instructions. The concentration and the purity of the RNA samples were checked using a Nanodrop spectrophotometer (Thermo Scientific, Waltham, MA, USA). The miRNA expression profiling was performed using Affymetrix GeneChip® miRNA 4.0 arrays (Affymetrix, Santa Clara, CA, USA) containing 30434 mature miRNAs. The mRNA expression profiling was performed using the Affymetrix GeneChip® Mouse Gene 2.0 ST Array containing 770,317 probes. With regard to miRNA expression profiling, the RNA was labelled using the FlashTag Biotin HSR (Genisphere, Hatfield, PA, USA) and was then hybridised to the miRNA array. After hybridization, staining and washing were performed according to the user guide. For mRNA expression profiling, the RNA was reverse transcribed to double-stranded cDNA, fragmented and labelled using the Biotin labelling kit, and then hybridized to the gene array as recommended. Standard Affymetrix array cassette staining, washing and scanning were then performed. In the last step for scanning the signals and analysing the data, Affymetrix® Expression Console Software (version 1.2.1) was used for the microarray analysis. Raw data (CEL files) were normalised at the transcript level by using a robust multi-average (RMA) method.

### Data analysis

Normalised data were analysed from each array. The fold change and the detection of p-values were used to screen the miRNAs and the mRNAs that showed significantly different expressions. Hierarchical clustering of the miRNAs and mRNAs with significantly different expressions was performed using the Cluster 3.0 software, and was visualised using MultiExperiment Viewer (www.tm4.org).

### Integrated analysis of the miRNA targets

DIANA database (http://www.microrna.gr/microT-CDS)^34^,^35^ and microRNA.org^36^ were used to predict the putative targets of differentially expressed 44 miRNAs. To improve the accuracy of the target prediction, we combined the microarray data of differentially expressed mRNA with the predicted targets of the differentially expressed 44 miRNAs. The intersecting gene set was subjected to the subsequent bioinformatic analysis.

### Bioinformatic analysis

To identify the functional pathways of intersecting genes and to uncover the miRNA-gene regulatory network on the basis of biological processes and molecular functions, we applied the Kyoto Encyclopedia of Genes and Genomes (KEGG, http://www.genome.ad.jp/kegg) enrichment analysis^37^,^38^. The list of the intersecting genes was matched onto the Database for Annotation, Visualisation and Integrated Discovery (DAVID) v6.7^39^,^40^, followed by the listing of the KEGG pathways with the p-value of the analysis.

### mirConnX

The web interface mirConnX^41^ (http://www.benoslab.pitt.edu/mirconnx) was used to generate mRNA-miRNA interaction networks. The normalised miRNA and mRNA microarray expression data were used as input files. We selected mouse-mm9 (NCBI37)) _20110812 for organism type. Gene symbol and ID were selected for the gene ID and the miRNA ID, respectively. In the analysis options section, we selected Pearson’s correlation as the association measure of choice. The regulation threshold for the minimum integrated regulation score was set at 0.5.

### MicroRNA quantitative real-time polymerase chain reaction (qRT-PCR)

Using the same total RNA samples, relative expression levels of the selected miRNAs (mmu-miR-3078-5p, mmu-miR-200c-3p, mmu-miR-496a-5p, mmu-miR-412-5p, mmu-miR-323-5p) were analysed after reverse transcription with specific RT primers (TaqMan® MicroRNA Assay, Applied Biosystems, Cheshire, UK) following the manufacturer’s guidelines. Fifty nanograms of the total RNA from the samples was used for the reverse transcription. The cDNA was amplified by the qRT-PCR with Universal TaqMan Mix (with no Amperase Ung) and miRNA-specific primers (Applied Biosystems), following the manufacturer’s protocol. Reactions were performed on the qRT-PCR machine, CFX connect (BioRad, Reinach, Switzerland). All reactions were performed in triplicate, and their relative abundances were analysed by normalising with respect to small nucleolar RNA (snoRNA). Two groups were compared according to their 2^−ddCt^ values, and the significance was calculated using a one-way analysis of variance (ANOVA), followed by Dunnett’s post hoc tests with the level of statistical significance set at P < 0.05.

## Additional Information

**How to cite this article**: Lee, S. *et al.* Integrated miRNA-mRNA analysis in the habenula nuclei of mice intravenously self-administering nicotine. *Sci. Rep.* **5**, 12909; doi: 10.1038/srep12909 (2015).

## Supplementary Material

Supplementary Information

## Figures and Tables

**Table 1:** Forty-four nicotine-responsive miRNAs in Hb.

	Chromosome map	Sequence	MHb fold-change	Detection *P*-value MHb control	Detection *P*-value MHb nicotine	LHb fold-change	Detection *P*-value LHb control	Detection *P*-value LHb nicotine
**Up regulated in MHb**								
** mmu-miR-3078-5p**	chr14:64591200–64591222 (+)	caaagccuagacugcagcuaccu	3.369	4.21E-08	2.46E-14			
mmu-miR-496a-3p	chr12:109739165–109739186 (+)	ugaguauuacauggccaaucuc	2.945	2.33E-11	1.16E-14			
** mmu-miR-200c-3p**	chr6:124718324–124718346 (−)	uaauacugccggguaaugaugga	2.803	1.34E-11	8.72E-16			
** mmu-miR-496a-5p**	chr12:109739132–109739152 (+)	agguugcccaugguguguuca	2.489	4.72E-13	8.72E-16			
mmu-miR-665-5p	chr12:109586331–109586356 (+)	aggggccucugccucuauccaggauu	2.446	3.24E-05	5.85E-09			
mmu-miR-1231-5p	chr1:135454661–135454683 (−)	ucugggcagagcugcaggagaga	2.242	8.72E-16	8.72E-16			
** mmu-miR-412-5p**	chr12:109743303–109743325 (+)	uggucgaccagcuggaaaguaau	2.160	8.72E-16	8.72E-16			
** mmu-miR-323-5p**	chr12:109712523–109712544 (+)	aggugguccguggcgcguucgc	2.111	4.91E-10	1.04E-12			
**Down regulated in MHb**								
mmu-miR-5132-5p	chrX:74023571–74023591 (−)	gcguggggugguggacucagg	0.485	1.32E-05	4.05E-05			
mmu-miR-669h-5p	chr2:10518174–10518197 (+)	augcauggguguauaguugagugc	0.478	4.92E-14	5.72E-11			
mmu-miR-467e-5p	chr2:10505731–10505752 (+)	auaagugugagcauguauaugu	0.418	1.17E-12	5.48E-11			
mmu-miR-673-3p	chr12:109572044–109572066 (+)	uccggggcugaguucugugcacc	0.385	3.44E-08	3.66E-06			
mmu-miR-193a-3p	chr11:79712009–79712030 (+)	aacuggccuacaaagucccagu	0.309	8.72E-16	7.49E-10			
mmu-miR-199b-5p	chr2:32318485–32318507 (+)	cccaguguuuagacuaccuguuc	0.299	5.33E-09	4.16E-08			
mmu-miR-467b-5p	chr2:10481256–10481276 (+)	guaagugccugcauguauaug	0.250	3.60E-14	8.39E-08			
**Up regulated in LHb**								
mmu-miR-375-3p	chr1:74900661–74900682 (−)	uuuguucguucggcucgcguga				10.22	1.64E-14	8.72E-16
** mmu-miR-200c-3p**	chr6:124718324–124718346 (−)	uaauacugccggguaaugaugga				6.395	1.87E-10	8.72E-16
mmu-miR-183-5p	chr6:30169711–30169732 (−)	uauggcacugguagaauucacu				3.221	9.57E-09	2.15E-12
mmu-miR-3074-1-3p	chr13:63301211–63301232 (−)	gauaucagcucaguaggcaccg				3.044	3.17E-06	4.71E-12
mmu-miR-23a-5p	chr8:84208527–84208548 (+)	gggguuccuggggaugggauuu				2.863	1.51E-05	7.04E-07
mmu-miR-1251-5p	chr10:92137190–92137210 (−)	acucuagcugccaaaggcgcu				2.521	8.94E-10	8.72E-16
** mmu-miR-496a-5p**	chr12:109739132–109739152 (+)	agguugcccaugguguguuca				2.300	2.90E-15	8.72E-16
** mmu-miR-412-5p**	chr12:109743303–109743325 (+)	uggucgaccagcuggaaaguaau				2.273	8.72E-16	8.72E-16
** mmu-miR-3078-5p**	chr14:64591200–64591222 (+)	caaagccuagacugcagcuaccu				2.254	2.03E-12	4.69E-15
** mmu-miR-323-5p**	chr12:109712523–109712544 (+)	aggugguccguggcgcguucgc				2.004	2.89E-12	8.72E-16
**Down regulated in LHb**								
mmu-miR-451a	chr11:78073186–78073207 (+)	aaaccguuaccauuacugaguu				0.458	8.72E-16	8.72E-16
** mmu-miR-223-3p**	chrX:96242884–96242905 (+)	ugucaguuugucaaauacccca				0.452	5.59E-10	2.69E-07
mmu-miR-322-3p	chrX:53054269–53054289 (−)	aaacaugaagcgcugcaacac				0.424	8.62E-12	8.90E-10
** mmu-miR-669c-3p**	chr2:10509359–10509380 (+)	uacacacacacacacaaguaaa				0.375	2.44E-12	3.74E-09
mmu-miR-325-3p	chrX:105379105–105379126 (−)	uuuauugagcaccuccuaucaa				0.355	1.03E-13	2.02E-11
mmu-miR-376b-5p	chr12:109723471–109723492 (+)	guggauauuccuucuaugguua				0.292	1.66E-13	4.64E-09
mmu-miR-374b-5p	chrX:103573112–103573133 (−)	auauaauacaaccugcuaagug				0.288	1.04E-14	5.72E-07
mmu-miR-712-5p		cuccuucacccgggcgguacc				0.280	1.13E-06	0.006707
**Opposite pattern**								
mmu-miR-721	chr5:136375777–136375797 (−)	cagugcaauuaaaagggggaa	0.428	1.08E-06	0.004189	2.315	0.00751	5.39E-06
mmu-miR-5621-3p	chr11:115795866–115795886 (+)	ugggcccuccagaccucaugc	0.446	0.002781	0.027158	2.130	0.014854	0.002771
mmu-miR-6996-5p	chr2:26470094–26470114 (−)	ugcacaggacagagcacaguc	0.200	0.000348	0.01303	1.928	0.000366	0.00028
mmu-miR-7012-5p	chr3:90270154–90270176 (+)	aaggagaggaguuggcagggacu	0.600	8.99E-07	1.02E-05	1.859	2.1E-06	3.08E-06
mmu-miR-467c-3p	chr2:10473977–10473998 (+)	auauacauacacacaccuauac	0.602	1.02E-09	4.08E-08	1.694	1.19E-06	6.05E-06
mmu-miR-6378	chr3:34922623–34922644 (−)	ugguucacgggaggacacacgc	0.662	8.94E-05	0.000766	1.685	0.053792	0.002375
mmu-miR-7683-3p	chr1:171641840–171641860 (−)	uggaaagguggaacacggaac	0.611	5.8E-06	0.000457	1.685	5.08E-05	1.65E-05
mmu-miR-210-5p	chr7:141221445–141221466 (−)	agccacugcccaccgcacacug	0.662	5.56E-05	5.3E-05	1.635	4.11E-05	1.58E-05
mmu-miR-3474	chr2:158638583–158638604 (+)	cccugggaggagacguggauuc	0.653	0.001264	0.005752	1.595	0.010695	0.000605
mmu-miR-6912	chr10:80609305–80609329 (+)	uacagggagggugcucaggcag	1.888	0.000729	0.000111	0.655	0.001865	0.053524
mmu-miR-7001-5p	chr2:93421980–93422002 (−)	aggcagggugugagcgugagcau	1.714	0.000279	4.04E-06	0.580	3.66E-06	6.7E-06
mmu-miR-542-5p	chrX:53049453–53049474 (−)	cucggggaucaucaugucacga	1.931	0.000668	5.92E-06	0.516	2.82E-07	9.35E-06
mmu-miR-6933-3p	chr11:118002324–118002343 (+)	agguguuuccucugccguca	2.464	0.003668	0.000377	0.455	3.79E-08	9.38E-06
** mmu-miR-223-3p**	chrX:96242884–96242905 (+)	ugucaguuugucaaauacccca	1.667	1.08E-06	1.52E-06	0.452	5.59E-10	2.69E-07
mmu-miR-6926-5p	chr11:74868168–74868189 (−)	ucaguggggugagggaugguga	1.770	0.000327	0.000154	0.450	2.05E-06	8.07E-06
mmu-miR-455-5p	chr4:63256867–63256888 (+)	uaugugccuuuggacuacaucg	2.216	3E-05	7.74E-06	0.416	2.8E-08	2.83E-05
** mmu-miR-669c-3p**	chr2:10509359–10509380 (+)	uacacacacacacacaaguaaa	2.141	9.64E-08	3.5E-08	0.375	2.44E-12	3.74E-09
mmu-miR-7053-3p	chr7:44538106–44538126 (−)	cuccugugucuccuuccccag	1.585	0.004188	0.00165	0.325	0.000253	0.011784

Forty-four microRNAs selected from the array data were shown. Each genomic locus within the chromosome and sequence of the miRNAs were represented. Fold-changes and detection p-values of the array were listed. In addition to the up- or down-regulated miRNAs in MHb and LHb, miRNAs that were oppositely regulated between MHb and LHb were also listed. The p cutoff value was P < 0.01 except for the opposite pattern. The p cutoff value of the opposite pattern was P < 0.05 except for the 2 microRNAs with the p-value of 0.053792 and 0.053524, respectively.

**Table 2:** List of 10 genes in the mRNA microarray which were oppositely changed by respective alteration of the targeting miRNA.

Gene symbol	Gene accession	MHb foldchange	LHb foldchange	Targeting miRNA	Description
Nlrp4a	ENSMUST00000068767	0.852	1.360	mmu-miR-669c-3p	NLR family, pyrin domain containing 4A
Zfp820	NM_029281	0.874	1.146	mmu-miR-223-3p	Zinc finger protein 820
Oas1a	NM_145211	0.821	1.137	mmu-miR-669c-3p	2′-5′oligoadenylate synthetase 1A
Prss55	NM_001081063	1.105	0.898	mmu-miR-3474	protease, serine, 55
Rfpl4b	NM_001177783	1.270	0.891	mmu-miR-721	Ret finger protein-like 4B
Cd69	NM_001033122	1.153	0.887	mmu-miR-721	CD69 antigen
Cenpi	ENSMUST00000081064	1.156	0.825	mmu-miR-467c-3p	centromere protein I
Klrb1c	NM_001159904	1.138	0.806	mmu-miR-3474	killer cell lectin-like receptor subfamilyB member1C
C87414	ENSMUST00000162964	1.155	0.767	mmu-miR-5621-3p	Expressed sequence C87414
Gm5724	ENSMUST00000148411	1.345	0.747	mmu-miR-3474	predicted gene 5724

Representative genes from the mRNA array data of which the alteration patterns were matched with the oppositely changed miRNAs. Gene symbol, accession number, fold change value of the alteration, targeting miRNA and description of the target genes are listed.
