# Supplementary material for: Integrated miRNA-mRNA analysis in the habenula nuclei of mice intravenously self-administering nicotine
Source: Sci Rep. 2015 Aug 11;5:12909. doi: 10.1038/srep12909 (PMC4531287; doi:10.1038/srep12909)
Supplement: Supplementary Information [file srep12909-s1.pdf]

Title: Integrated miRNA-mRNA analysis in the habenula nuclei of mice intravenously self-administering nicotine

Authors' names: Sangjoon Lee[1], Jiwan Woo[1,2], Yong Sik Kim[4], Heh-In Im[1,2,3*]

Affiliations:

[1]Center for Neuroscience, Brain Science Institute, [2]Research Animal Resource Center, Korea Institute of Science and Technology, Hwarangno 14-gil 5, Seongbuk-gu, Seoul, Republic of Korea, [3]Neuroscience Program, Korea University of Science and Technology, 217 Gajungro, Yuseong-gu, Daejeon, Republic of Korea. [4]Department of Pharmacology, College of Medicine, Seoul National University, Seoul, Republic of Korea.

*Correspondence to him@kist.re.kr

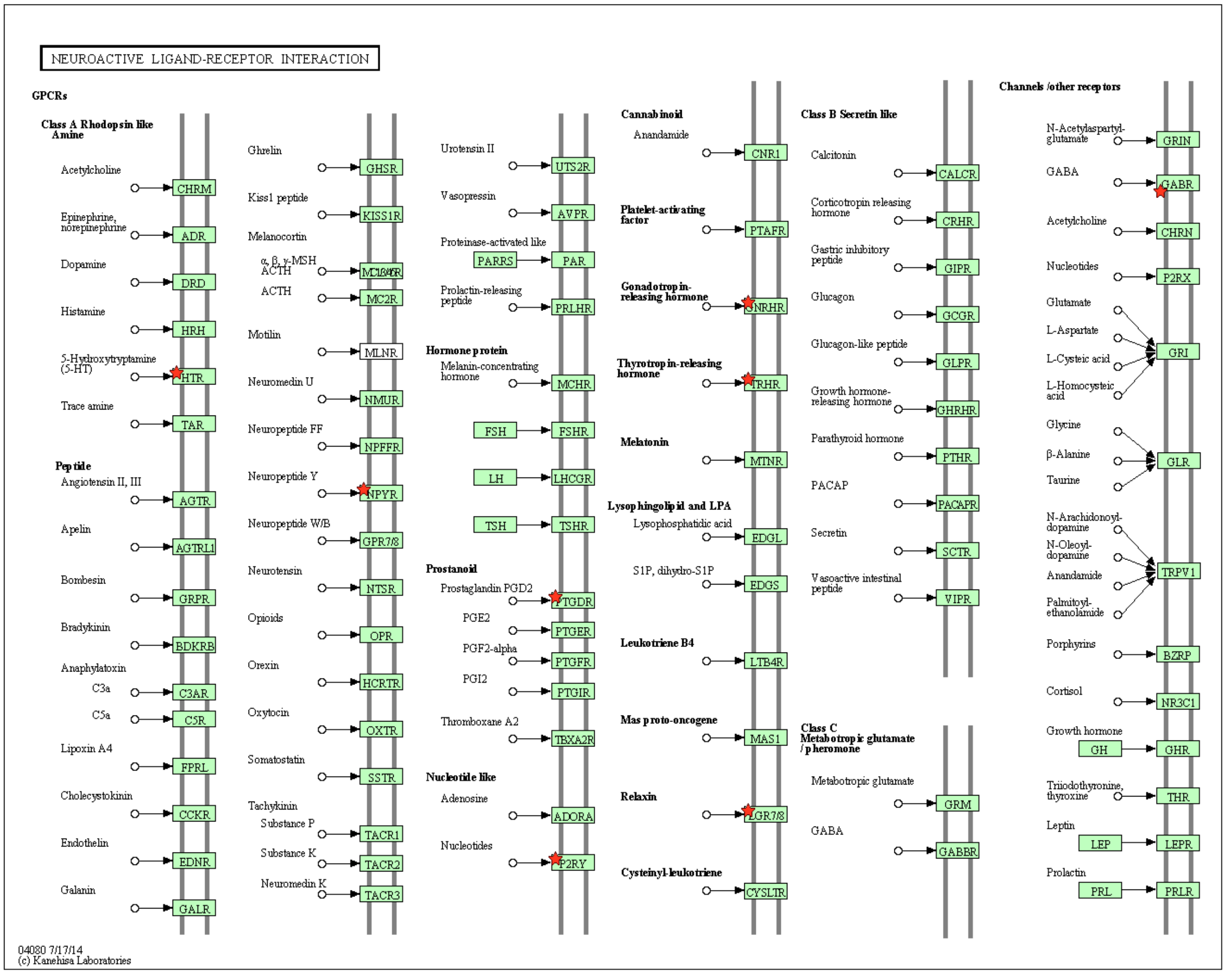

Supplementary Figure S1. KEGG metabolic pathways of neuroactive ligand-receptor interaction. Red marks represent up-regulated mRNAs targeted by down-regulated miRNAs in LHb of nicotine SA group.

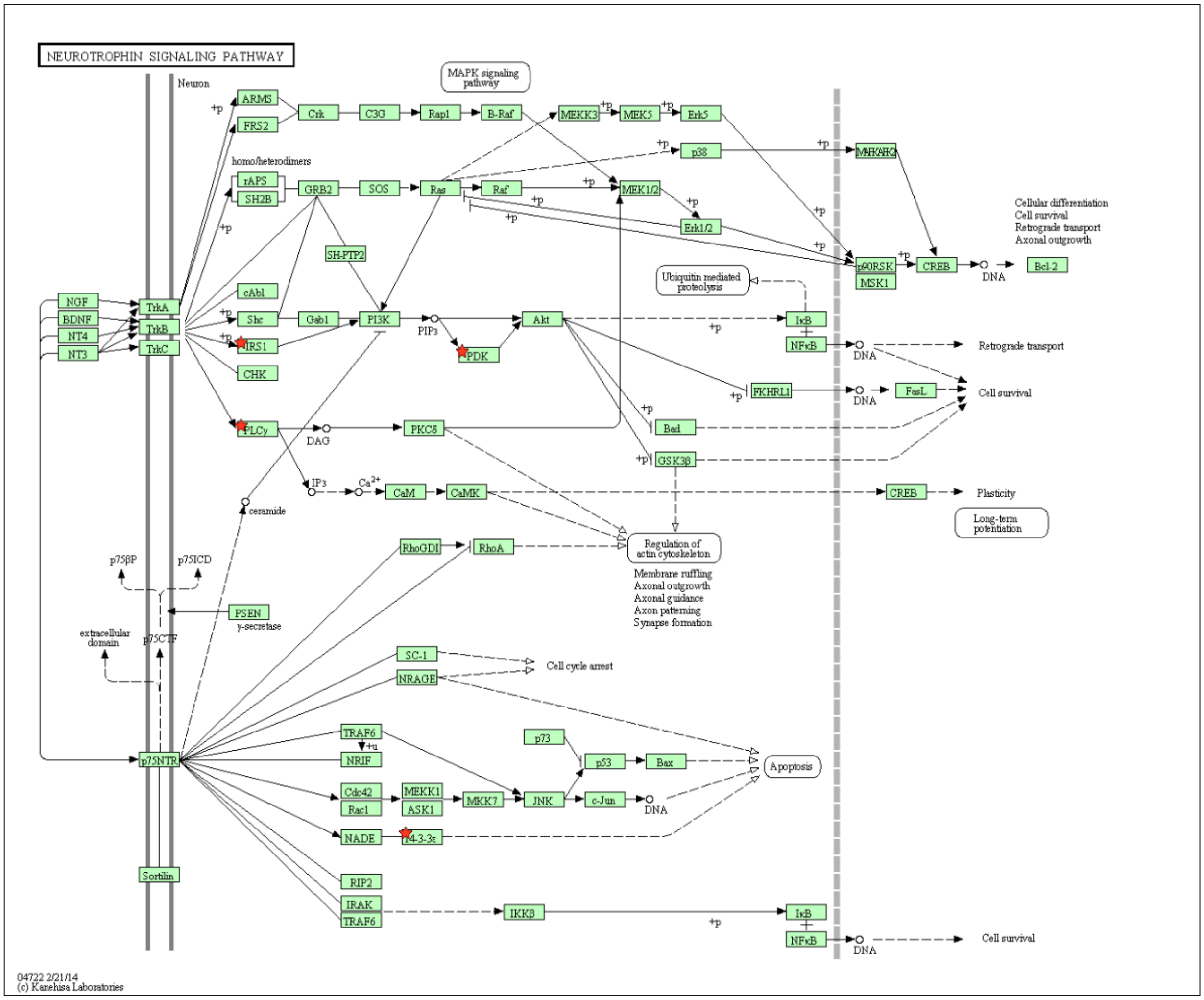

Supplementary Figure S2. KEGG metabolic pathways of neurotrophin signaling pathway. Red marks represent down-regulated mRNAs targeted by up-regulated miRNAs in MHb of nicotine SA group.

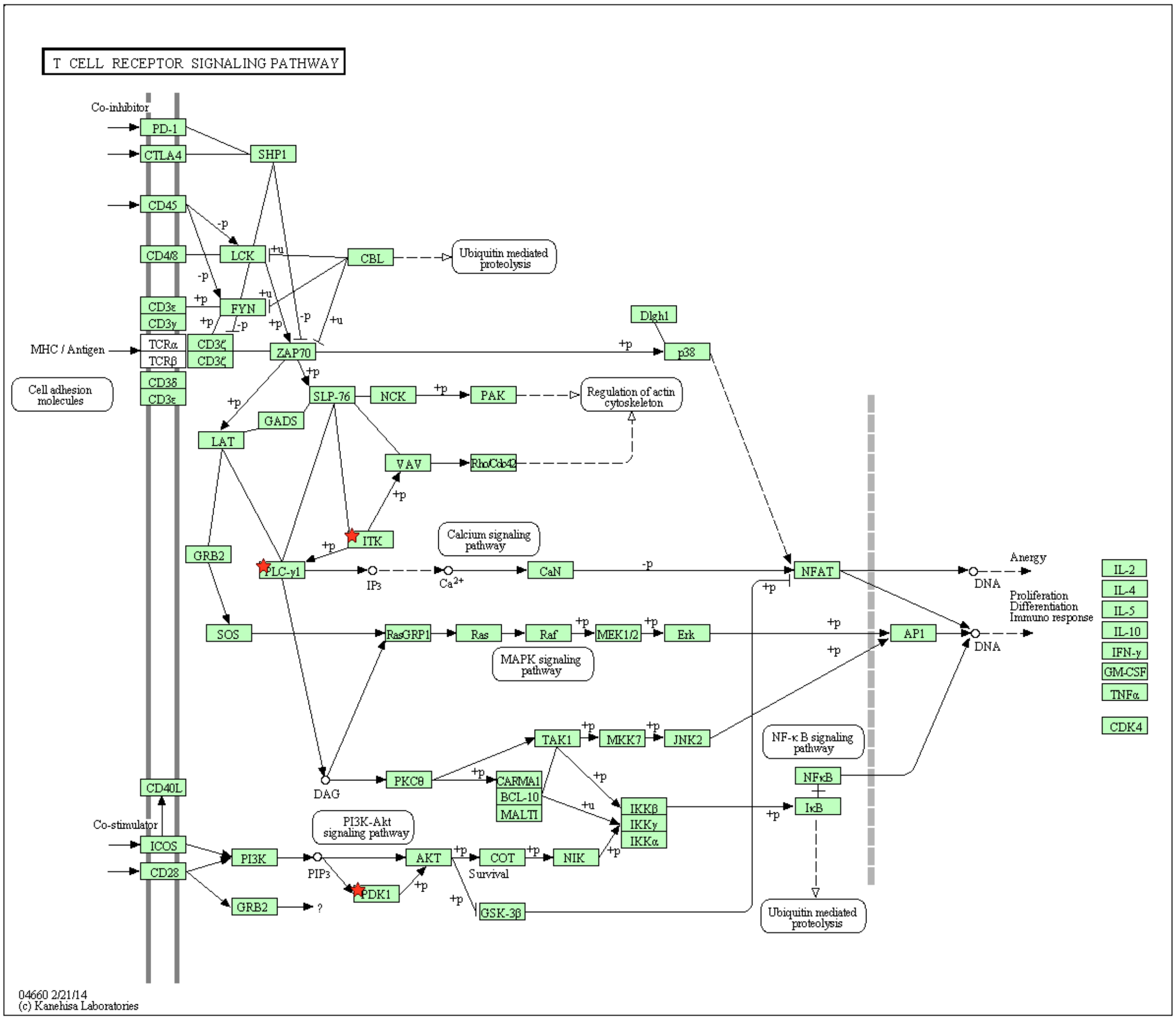

Supplementary Figure S3. KEGG metabolic pathways of T cell receptor signaling pathway. Red marks represent down-regulated mRNAs targeted by up-regulated miRNAs in MHb of nicotine SA group.

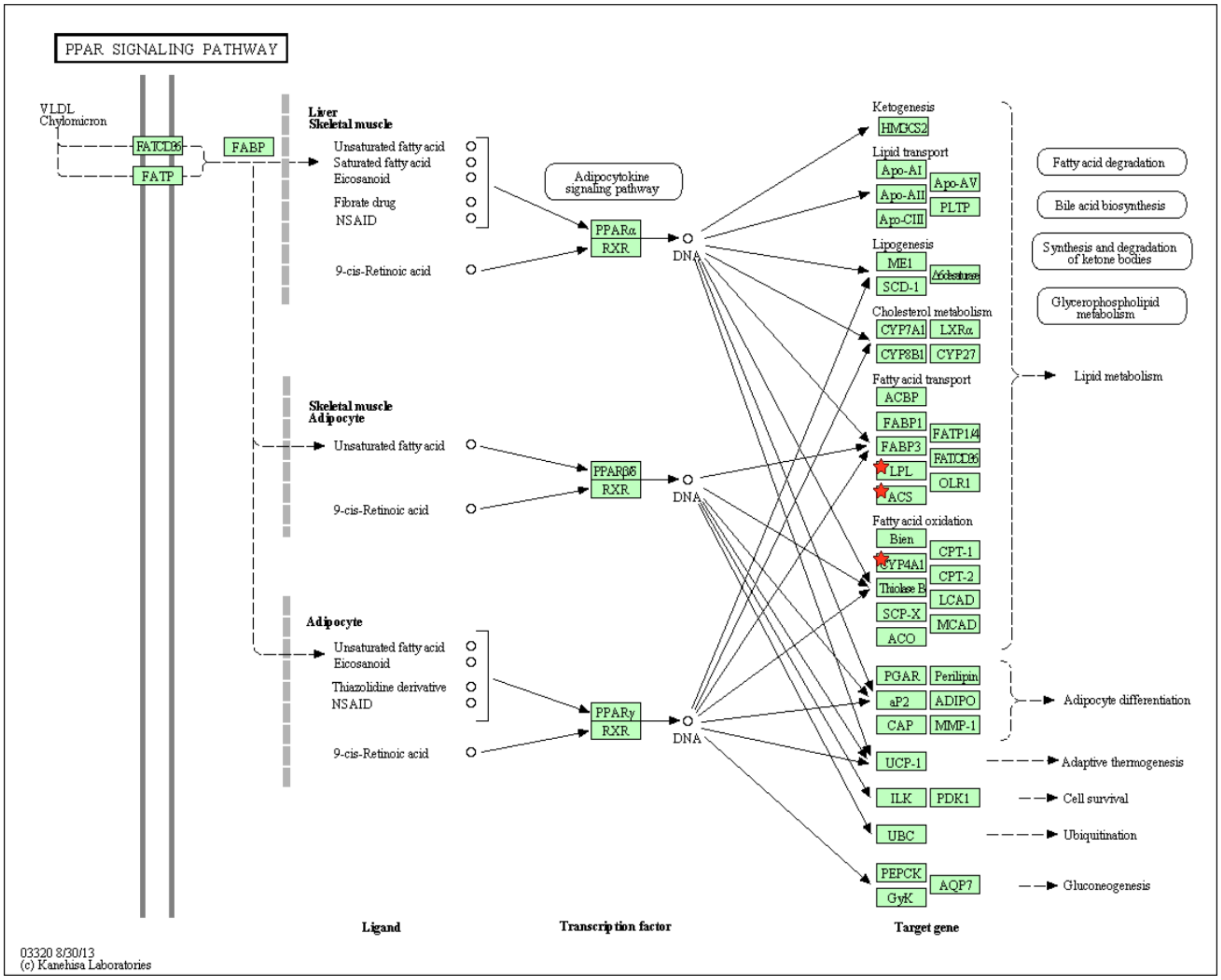

Supplementary Figure S4. KEGG metabolic pathways of PPAR signaling pathway. Red marks represent down-regulated mRNAs targeted by up-regulated miRNAs in LHb of nicotine SA group.

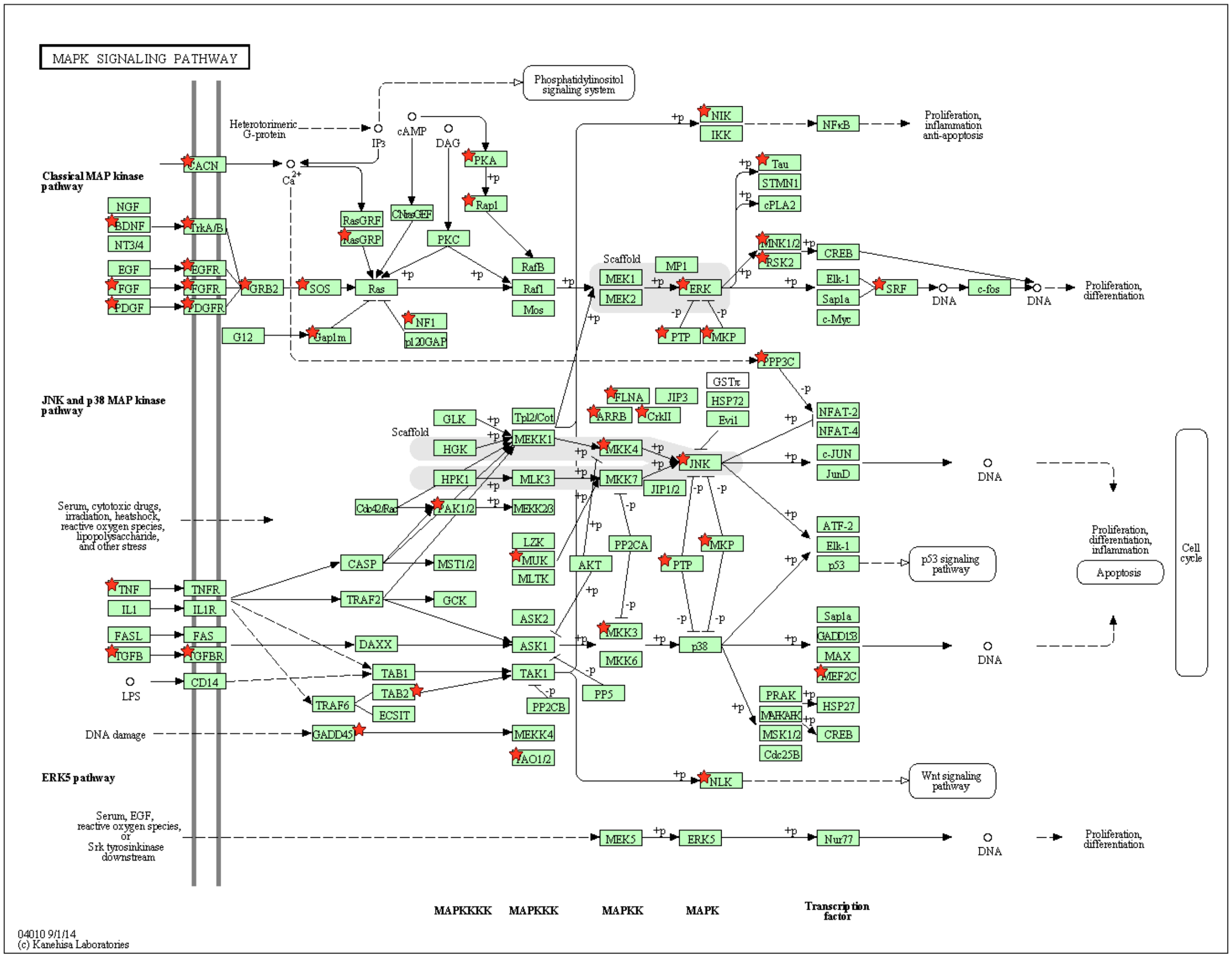

Supplementary Figure S5. KEGG metabolic pathways of MAPK signaling pathway. Red marks represent altered mRNAs targeted by nicotine-responsive miRNAs in nicotine SA group.

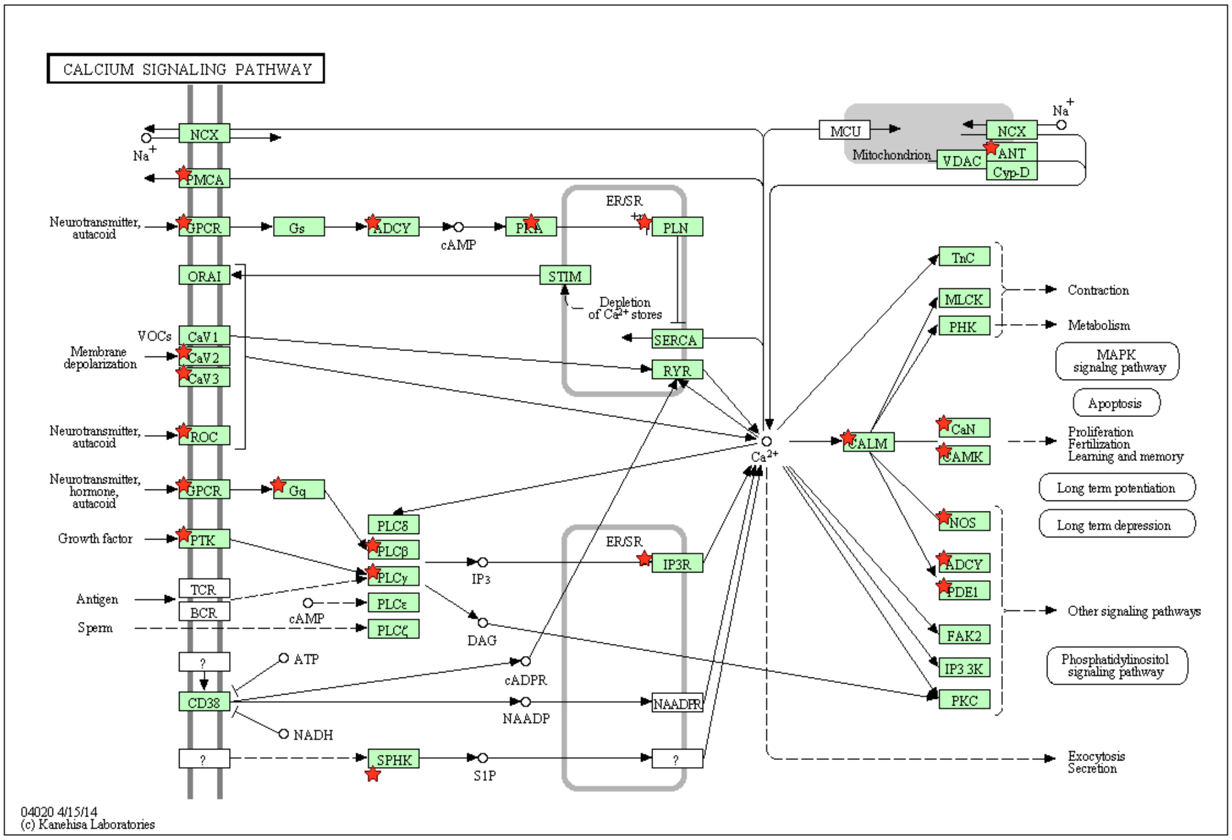

Supplementary Figure S6. KEGG metabolic pathways of calcium signaling pathway. Red marks represent altered mRNAs targeted by nicotine-responsive miRNAs in nicotine SA group.

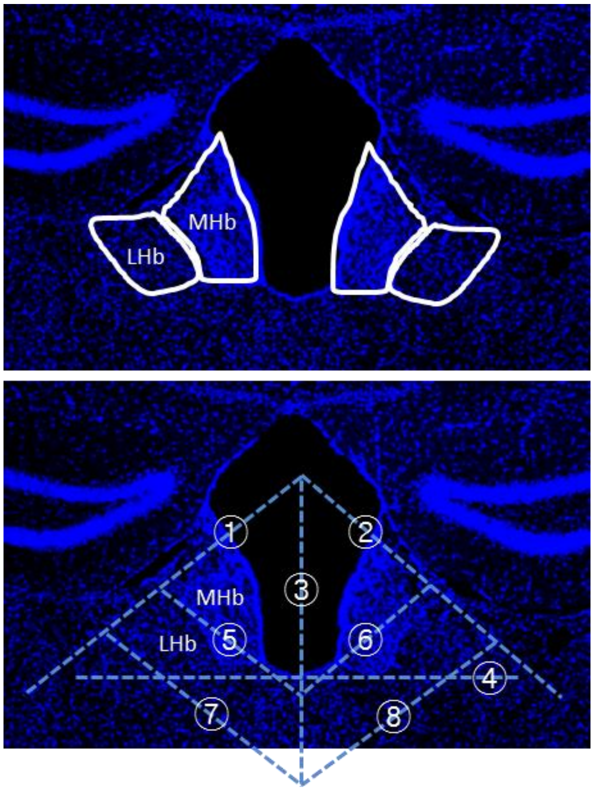

Supplementary Figure S7. Coronal section of the mouse brain representing the Hb region and conceptual scheme of the micro-dissection of the MHb and LHb. Every 100 µm section was cut in the numerical order in the lower panel and 2 pieces of section were collected from the MHb and the LHb, respectively.

Down-regulated genes in MHb	Up-regulated genes in MHb	Down-regulated genes in LHb	Up-regulated genes in LHb	
Nop56	Casp4	Gm13212	Il1b	Gm3676
Zfp26	Kcnq5	Cbln3	Gm595	H2-Aa
Srsf6	Gm4951	Otc	Tbx19	BC025446
Olfr715	Gpr141	Isl2	F5	Gabrp
Tmprss11bnl	Acer2	LOC380994	Kifc1	Lipm
Arhgap26	P2ry10	Fndc3c1	Abca13	Srcrb4d
Krtap31-1	Serpinc1	Psg25	Gdf6	Gm14446
Rhox2d	Gm6531	Mpp7	Rtkn2	Mup8
Car3	Slc26a7	Nsl1	Gmcl1l	Gm4070
Gm5077	Slc6a5	Chsy1	Tacstd2	4932411G14Rik
Gm904	Gm4902	2300005B03Rik	Mapkbp1	Thbs1
Gm12511	Gm17365	2200002J24Rik	Mrgprd	Mbnl3
Gtf2a1	Ssxb10	Cyp3a25	Bex6	Ms4a6c
BC052688	Lyve1	Gpr183	Adamts5	Exoc5
Adam25	Ang4	Dhrs7c	Gm5141	U2af1l4
Ugt3a2	Ccr3	4930503B20Rik	Gm13219	Rhox3c
Srpx	Gm5420	Slain1	En1	4933411K16Rik
A630038E17Rik	AF067063	Cd69	Mlf1ip	Gprasp2
Klri2	Psg27	4930459I23Rik	4930449C09Rik	Speer4e
Cfhr1	Gbp3	Slc25a31	Gcnt3	Mup19
Nlrp4f	Acsm5	Rad54b	Tmprss11bnl	Ogn
Heatr5a	Piwil1	Cpxm2	BC039771	Oas1a
Cyp4a32	Dmp1	Iqch	Osmr	Gsdmd
BC024659	Scara5	Rex2	Cdh19	Gm15128
Zfp820	Sprr2k	Nlrp9c	Dach1	Gm8439
Depdc1a	2900041M22Rik	Gsdmc2	Hspb8	Penk
4930442J19Rik	Sectm1b	B020018G12Rik	Zfp711	Fmo2
Upb1	Pnpla1	Mrgprb1	Chi3l3	Atg9b
Ttc30b	Alox5	Gm14374	Ssxb9	Phex
Sel1l2	Timm8a1	1700074P13Rik	Olfr1033	Kctd8
Il12a	6030498E09Rik	Vmn1r90	Sly	Hrnr
Akr1c19	Mcpt8	5430427M07Rik	Csn1s1	Lilrb4
Psd3	Dtl	Lce1g	Gbp5	Nlrp4c
1700095A21Rik	Dppa5a	C87414	Gm1698	Glyat
Has2	Rbmy1a1	Ssx9	Nlrp4f	Cldn1
Tmem92	Apol10a	6030498E09Rik	Trhr	Gvin1
Pcdhga11	Clec2g	Fbxo48	Gm3643	Mup15
Ncapg	Il21r	Catsperg2	Kif4	Ifitm3

Cts8	Agtr2	Pfpl	Lilrb3	Bach2
Khdc1a	G630055G22Rik	BC034902	5330437I02Rik	Krtap6-1
Rabggtb	Slfnl1	Seh1l	Defb11	Timd2
Seh1l	Zbp1	Gpr82	Tceal7	Trim54
Ifna11	Fam169b	Hoxa1	Ptgs2	Cdkn1a
2610305D13Rik	Lpin2	Gpr174	Gm13119	Eda2r
Gm5868	2610206C17Rik	5430425J12Rik	Ebf2	4930534B04Rik
Vmn2r28	Ifi203	Wfdc16	Gm609	Sall3
Odz1	Igll1	Ssxb10	5830428M24Rik	Krt84
Pdk1	Btn2a2	Obox5	Clca2	Cldn2
Hnrnpk	Apof	Vmn2r66	Xpnpep2	Bend4
Prl8a1	Dach2	Cpxcr1	Gm10436	Mustn1
Nlrp4a	Il10	Prss2	Il28ra	Gad2
Gm14374	Rgs1	Psg29	Doxl2	Htr4
Lipk	Cypt2	H28	1700010M22Rik	Slc6a14
Mstn	Triml1	1700003P14Rik	Dennd1b	Klk1b22
1700042B14Rik	Skint8	Tmem210	Gnrhr	Thbd
Eddm3b	Krr1	Eddm3b	Gzmb	Krtap16-5
B020018G12Rik	Cckar	Vmn1r4	Zfp708	1500003O03Rik
Ttc23l	Col4a6	Syne1	Csn2	H2-T23
Abat	Olfr1502	Obox3	Dut	Flt4
Mtus1	Prss43	Gm7714	Ifi44	4930579C15Rik
Lrat	4933436I01Rik	Prl8a1	Klra9	Slc16a9
Yap1	Zfp735	Tnfsf11	Kir3dl1	Amica1
Macc1	C87977	Acsm2	Cyp2f2	5430401F13Rik
Itk	Klrb1b	Msn	Postn	1700042B14Rik
Gpr82	Crxos1	Drd5	Cyp2b19	Csta
Lypla1	Dut	Dmp1	Nr4a2	She
Tgds	Opn4	4930442J19Rik	H2-M10.5	Klk1b9
Pvrl4	Lefty2	Pvrl4	1700025D23Rik	Stil
Ddx26b		Il21r	Serpinb6b	Mup13
Ywhag		Ncapg	Ankrd22	Klk1b21
Lrrc6		Xcr1	Zfp820	Tiam1
Gm15114		Ckap4	Klhl14	Glyctk
Wfdc16		1700034J05Rik	Serpinb1c	Zbp1
Irs4		Ugt2b35	A730082K24Rik	Cd200r2
BC034902		Olfr701	Catsperb	Eif2ak2
Plcg1		Gm10389	Ptgdr	Psg17
Ppp2ca		Il23a	Tnfsf18	D5Ertd577e
Lce1c		Gsdmc3	4933406F09Rik	Prl7b1

Zeb2		Ccdc68	Cyp2c44	Acss1
Gpr119		Il10	Ppp2ca	Zdhhc20
Vat1l		Hand2	Ppp1r3a	Rgs18
Naa40		Gucy2f	Sim1	Kcnh8
Ccdc110		Pdc	Shisa3	Odz1
Paxip1		Lilra5	Lgals12	Emp1
Psg25		Hpse	9430076G02Rik	Klrc2
Ubiad1		Abca14	Isl1	Taf1d
Actg1		Hyal6	Il7	Cd200r4
Gm13102		Hist1h2ac	Pax3	Gngt1
		9530053A07Rik	Cd55	4930519F16Rik
		Gm5946	Dio3	Ms4a4b
		Rgs13	Fas	Pramel3
		Dpp4	Lrrtm4	Plagl1
		Lpl	Rnf17	Gm12657
		C130060K24Rik	Rsl24d1	Fndc3a
		Pof1b	Olfr156	Dclre1b
		Sfrs18	Gm6150	Krtap14
		Gm12603	Qrfpr	Slc5a7
		Rtp1	Dcaf12	Nlrp9b
		Myf6	Gm4956	Chrnb3
		Gm5531	Alpi	Krtap3-2
		Lypla1	Meis2	Ch25h
		Arl8b	Lpl	Irgm2
		Akr1c20	E330016L19Rik	2610305D13Rik
		Klrb1a	A830049A06	Gm3414
		Nlrp1c	Cenpk	Aldoart1
		Klrb1c	Zbbx	Vmn2r42
		P2ry10	Rxfp2	Tyrp1
		Slc17a3	Ugt2b1	Mmp8
		Pdilt	Wdr52	BC106179
		Cyp4a14	Gm11426	Gm13242
		Cstf2	Pomc	P2ry4
		Abpg	Hdx	Fry
		Fam175b	Gm3259	Gm5114
		Klrk1	C130079G13Rik	Klri2
		Apol10a	Cyyr1	Clec4a2
		Matn3	Iigp1	Gbp3
		1700065I16Rik	Gm13084	Mbd3l2
		Gm3279	Gm2381	Vat1l

		Capn13	Riok2	Ms4a4c
		Akr1c14	Defa22	4833417C18Rik
		Pde7a	Ifitm2	Kcna5
		Tnp2	Spinkl	Sntn
		Aqp8	Skint4	Pou4f1
		Ear6	Serpina3f	Gm5127
		4930471C06Rik	Tmprss11d	Klhl31
		4933413J09Rik	5530400C23Rik	Ccdc147
		Slc16a5	Krt18	Gtsf1
		Dact1	Mup10	Xlr4b
		A330050B17Rik	Rhox3f	Lyve1
		1700123I01Rik	Tmem184a	Zim1
		Ppih	Lefty2	Msh4
		Igh-VJ558	Klk1b27	Cyp2c67
		Zfp202	Rb1	A930004D18Rik
		Gm4894	Gm4937	Hist1h2bp
		Speer4a	A630033H20Rik	Efhb
		Defb6	Gpr83	Cyp2c39
		Tmem45a	Lbp	Htr2a
		Cyp4a12a	Mum1l1	Iltifb
		Ccrl2	Xirp2	Sec16b
		Rdh1	Slc22a14	4930432K09Rik
		Gm5643	Cst10	E230016K23Rik
		Awat2	C130026I21Rik	Gimap8
		Casc5	Mup7	Zfp366
		Cabyr	Pilra	Gm5087
		1700003E24Rik	Zfp735	Ly6c1
		Acsl6	Krt71	Dsg2
		Luzp4	Tac2	Gm606
		Klra17	Serpinb3c	Tmc3
		Gm15080	Mtpap	Tmem200a
		1700025E21Rik	Akr1b7	Zxda
		Skint2	Cyp2j5	Cytip
		Serpinb3a	Phyhipl	1810011O10Rik
		Tfap2b	Trex2	Slc25a13
		Synj2bp	Ms4a6b	Pf4
		Gm11128	Hgd	3110003A17Rik
		Il1f8	Irgm1	Cmya5
		Gm13078	Ctla2a	Chrna3
		Prpmp5	S100a4	Golt1a

		Pramef12	Nlrp2	Hsd3b2
				Serpinb10
				Gdnf
				Scml2
				Sh3tc1
				Cntnap3
				Krt82
				Ppp3r2
				Casc1

Supplementary Table S1. 605 genes matched between the list of predicted targets and the mRNA microarray data are shown. Up- or down-regulated genes in the MHb, LHb are represented, respectively.
